# Detoxification of Aflatoxin B1 by a Potential Probiotic *Bacillus amyloliquefaciens* WF2020

**DOI:** 10.3389/fmicb.2022.891091

**Published:** 2022-05-10

**Authors:** Guojun Chen, Qian’an Fang, Zhenlin Liao, Chunwei Xu, Zhibo Liang, Tong Liu, Qingping Zhong, Li Wang, Xiang Fang, Jie Wang

**Affiliations:** ^1^Guangdong Provincial Key Laboratory of Food Quality and Safety, Guangdong Provincial Key Laboratory of Nutraceuticals and Functional Foods, College of Food Science, South China Agricultural University, Guangzhou, China; ^2^Guangdong Moyanghua Grains and Oils Co., Ltd., Yangjiang, China

**Keywords:** aflatoxin B1, *Bacillus amyloliquefaciens*, *Aspergillus flavus*, genome sequence, Ames test, *Caenorhabditis elegans*

## Abstract

Microbial degradation is considered as an attractive method to eliminate exposure to aflatoxin B1 (AFB1), the most toxic mycotoxin that causes great economic losses and brings a serious threat to human and animal health, in food and feed. In this study, *Bacillus amyloliquefaciens* WF2020, isolated from naturally fermented pickles, could effectively degrade AFB1 ranging from 1 to 8 μg/ml, and the optimum temperature and pH value were 37–45°C and 8.0, respectively. Moreover, *B. amyloliquefaciens* WF2020 was considered to be a potential probiotic due to the synthesis of active compounds, absence of virulence genes, susceptibility to various antibiotics, and enhanced lifespan of *Caenorhabditis elegans*. Extracellular enzymes or proteins played a major role in AFB1 degradation mediated by *B. amyloliquefaciens* WF2020 into metabolites with low or no mutagenicity and toxicity to *C. elegans*. AFB1 degradation by the cell-free supernatant was stable up to 70°C, with an optimal pH of 8.0, and the cell-free supernatant could still degrade AFB1 by 37.16% after boiling for 20 min. Furthermore, *B. amyloliquefaciens* WF2020 caused a slight defect in fungal growth and completely inhibited AFB1 production when co-incubated with *Aspergillus flavus*. Additionally, *B. amyloliquefaciens* WF2020 suppressed the expression of 10 aflatoxin pathway genes and 2 transcription factors (*alfR* and *alfS*), suggesting that *B. amyloliquefaciens* WF2020 might inhibit AFB1 synthesis in *A. flavus*. These results indicate that *B. amyloliquefaciens* WF2020 and/or its extracellular enzymes or proteins have a promising potential to be applied in protecting food and feed from AFB1 contamination.

## Introduction

Aflatoxins, a group of mycotoxins produced mainly by *Aspergillus flavus* and *Aspergillus parasiticus*, contaminate about 60–80% of food and feed around the world (Lee and Ryu, 2017; Eskola et al., 2019), and are also perceived as a severe threat to human health due to their hepatotoxicity, nephrotoxicity, immunotoxicity, etc. (Silvia et al., 2018). Among the 20 types of aflatoxins identified, aflatoxin B1 (AFB1) is the most toxic, mutagenic, and carcinogenic to both humans and livestock and is classified as a group-1 carcinogen by the International Agency for Research on Cancer (IARC, 1993). AFB1 contamination in crops has become a widespread problem, and considerable investigations have been directed at finding methods, such as physical, chemical, and biological methods, to prevent its toxicity.

Microbial degradation was considered as an attractive method due to its specificity, efficiency, environmental friendliness, protection of the quality and flavor of food, and feasibility of the processes when applied in industries (Mishra and Das, 2003; Wu et al., 2009). In the last decade, beneficial microorganisms substantially were found to be capable of reducing AFB1 in contaminated media, including Actinobacteria (e.g., *Brachybacterium* sp., *Rhodococcus*, *Streptomyces*, *Nocardia*, and *Mycobacterium*), Bacillus (e.g., *Bacillus*, *Lysinibacillus*, *Streptococcus*, and *Staphylococcus*), -Proteobacteria (e.g., *Enterobacter* sp., *Klebsiella*, *Pseudomonas*, and *Brevundimonas*), Ascomycota (e.g., *Aspergillus*, *Alternaria*, *Neurospora*, and *Trichoderma*), Basidiomycota (e.g., *Pleurotus*), Zygomycota (e.g., *Mucor*, *Rhizopus*, and *Absidia*), etc. (Verheecke et al., 2016). However, bacteria have more applications for AFB1 remediation due to some advantages such as more elimination within a shorter time and producing no pigments (Laciakova et al., 2008), and among them, *Bacillus* becomes an attractive candidate because of its high tolerance to various environmental stresses and application as a kind of potential probiotics (Yan et al., 2017). For instance, AFB1 was reduced by 92.1% by *Bacillus shackletonii* L7 for 72 h (Liang et al., 2017), 85.61% by *Bacillus subtilis* UTBSP1 for 96 h (Farzaneh et al., 2012), 91.5% by *Bacillus velezensis* DY3108 for 96 h (Shu et al., 2018), 94.70% by *Bacillus licheniformis* CFR1 for 72 h (Rao et al., 2016), and 100% by *Bacillus* TUBF1 for 72 h (El-Deeb et al., 2013). Although more and more *Bacillus* were reported to degrade AFB1, few studies have performed the safety assessment of selected strains. In addition, the narrow working temperature range and unsuitability for the processing environment in AFB1-degrading bacteria reported previously also become challenges in commercial applications. Therefore, it is still worth exploring safe bacteria, including *Bacillus*, which are suitable for food and feed processing and detoxify AFB1 into less toxic metabolites with excellent degradation efficiency and wide temperature ranges in the future.

*Bacillus amyloliquefaciens*, which was ubiquitously found in various environments, including food, plants, animals, soil, and aquatic environments, was reported as a potential probiotic due to its strong antimicrobial activity, the synthesis of bioactive compounds, including peptides and exopolysaccharides, its survival in gastrointestinal conditions, etc. (WoldemariamYohannes et al., 2020; Ngalimat et al., 2021). Moreover, *B. amyloliquefaciens* could be a multifunctional microbe and potentially applied in the animal food and feed industry and in functional food processing due to the improvement in the functional, sensory, and shelf life of end products and the production of several enzymes, including -glutamyl transpeptidase pectinase, xylanase, β-glucosidase, and amylase, which can hydrolyze complex compounds, including insoluble proteins, carbohydrates, fibers, hemicellulose, and lignans, and then increase the digestion and absorption of nutrients from food and feed and form novel functional and bioactive compounds (WoldemariamYohannes et al., 2020; Chen et al., 2021). In addition, some strains of *B. amyloliquefaciens*, such as *B. amyloliquefaciens* UTB2, UNRC52, and UNRCLR, could suppress AFB1 synthesis (Bluma and Etcheverry, 2006; Siahmoshteh et al., 2018). However, except for *B. amyloliquefaciens* S8C, Y1-B1, SWUN-TP23, SG-16, and HSP-5 (Xu et al., 2015; Guo et al., 2017; Wang J. et al., 2018; Ali et al., 2021; Zhang et al., 2021), little was known about the AFB1 degradation potential of *B. amyloliquefaciens* as well as the molecular mechanism of the loss in AFB1 production. Here, the AFB1-degrading bacterium in naturally fermented pickles was isolated and identified as *B. amyloliquefaciens* (WF2020), and the toxicities of the strain and its AFB1 degradation products were also assessed based on sequenced genome information, antibiotic susceptibility, the changes in the lifespan of *Caenorhabditis elegans*, and Ames mutagenicity. Moreover, the effects of cultivation conditions on AFB1 degradation mediated by *B. amyloliquefaciens* WF2020 and its active components were investigated by measuring the concentrations of residue AFB1 in media with different temperatures, pH values, and metal ions. Lastly, the effects of *B. amyloliquefaciens* WF2020 on the fungal growth and synthesis of AFB1 were investigated when *B. amyloliquefaciens* WF2020 was co-incubated with *A. flavus*, a producer of aflatoxins. The results indicated that *B. amyloliquefaciens* WF2020 is a potential probiotic applied in the protection of food and feed from AFB1 contamination.

## Materials and Methods

### Isolation of Potential Aflatoxin B1-Degrading Bacteria From Fermented Foods

About 1 g of fermented food was mixed with 10 ml of sterile saline and then diluted to 10^–3^, 10^–4^, 10^–5^, 10^–6^, and 10^–7^ levels. All dilutions were spread evenly on coumarin medium (CM: 1% coumarin, 0.025% KH_2_PO_4_, 0.1% NH_4_NO_3_, 0.1% CaCl_2_, 0.025% MgSO_4_⋅7*H*_2_O, 0.0001% FeSO_4_, and 1.5% agar) and cultured at 37°C for 4 days. Single colonies were isolated and transferred to fresh CM plates three times. Colonies growing on CM plates were selected and tested for AFB1 degradation.

### Aflatoxin B1 Degradation in Liquid Culture

Overnight cultured bacterial cells were diluted to an optical density at 600 nm (OD_600_) of 0.01 with fresh Luria-Bertani (LB) medium, and then AFB1 purchased from J&K Scientific (Beijing, China) was added into 1 ml of dilution for a final concentration of 2 μg/ml. Sterile LB medium with AFB1 was used as the control. After 3-day incubation at 37°C by shaking at 180 rpm, the supernatant was extracted using chloroform according to previous reports (Guan et al., 2008), and the chloroform fractions were evaporated and dissolved using dimethyl sulfoxide (Sigma-Aldrich, St. Louis, MO, USA). The redissolved solution was filtered using the 0.22-μm pore filter (Merck-Millipore, Darmstadt, Germany) and stored at −20°C for high-performance liquid chromatography (HPLC) detection. About 94–96% of AFB1 could be recovered from the liquid culture using chloroform extraction.

### Quantification of Aflatoxin B1 by High-Performance Liquid Chromatography

Aflatoxin B1 was analyzed by HPLC according to the procedure reported by Fang et al. (2020). The percentage of AFB1 degradation was calculated using the following formula: the percentage of AFB1 degradation = (1 − *C*_*a*_/*C*_*b*_) × 100%, where *C*_*a*_ and *C*_*b*_ are the concentration of remaining AFB1 in the sample and total AFB1 in the control sample, respectively.

### Analysis of Aflatoxin B1 Metabolites by HPLC-Quadrupole-Time-of-Flight-Mass Spectrometry

Aflatoxin B1 metabolites were extracted with chloroform after a 72-h incubation of AFB1 degrading bacterium in LB medium with 2 μg/ml of AFB1 and analyzed by HPLC-Q-TOF-MS according to the procedure described by Fang et al. (2020). Extractions from the AFB1-degrading bacterium in LB and sterile LB media with AFB1 were used as controls.

### Genome Sequencing and Analysis

Genomic DNA was extracted using Wizard^®^ Genomic DNA Purification kit (Promega, Beijing, China) according to the manufacturer’s protocol and sequenced using a combination of PacBio RS II Single Molecule Real Time (SMRT, Pacific Biosciences, MenloPark, CA, United States) and Illumina sequencing platforms (Hiseq X Ten; Illumina, San Diego, CA, United States). The PacBio reads and Illumina reads were used to assemble the complete genome sequence into a contig using the hierarchical genome assembly process (HGAP) and CANU (Version 1.7^1^). The last circular step was manually checked and finished, generating a complete genome with seamless chromosomes and plasmids. Finally, error correction of the PacBio assembly results was performed with Illumina reads using Pilon. Sequence data were deposited at the US National Center for Biotechnology Information (NCBI) under accession number CP092778.

The coding sequences (CDSs) were predicted with Glimmer (Version 3.02^2^) and annotated from the databases of Non-Redundant (NR Protein Sequence Database), Swiss-Prot, Pfam, Gene Ontology (GO), Clusters of Orthologous Group (COG), and Kyoto Encyclopedia of Genes and Genomes (KEGG) using sequence alignment tools such as Basic Local Alignment Search Tool (BLAST, Version 2.3.0^3^), Diamond (Version 0.8.3^4^), and HMMER (Version 3.1b2^5^), and annotations were obtained from the best-matched subjects (*E*-value < 10^–5^) for gene annotation. All data were analyzed on the free online Majorbio Cloud Platform^6^.

### Antibiotic Susceptibility

Disk diffusion susceptibility tests were conducted according to the procedure reported by the National Committee for Clinical Laboratory Standards. Briefly, a bacterial dilution (OD_600_ = 0.01) was spread on the Mueller-Hinton agar (MHA: 0.2% beef dehydrated infusion, 1.75% casein hydrolyzate, 0.15% starch, and 2% agar) plates and the disks with 2 μg lincomycin, 5 μg ciprofloxacin or rifampin, 10 μg gentamicin, streptomycin, ampicillin penicillin, imipenem, or norfloxacin, 15 μg erythromycin, or 30 μg tetracycline, cefalexin, kanamycin, chloramphenicol, or vancomycin were put on the plates. After 12 h of incubation at 37°C, the diameters of the inhibition zones were recorded.

### Assay for the Lifespan of *Caenorhabditis elegans*

Lifespans were monitored as described previously (Donato et al., 2017). Briefly, L4 worms of *C. elegans* N2 were grown on a nematode growth medium (NGM) agar plate seeded with *Escherichia coli* OP50 at 20°C and treated with alkaline hypochlorite to collect embryos. Embryos were cultivated to obtain a synchronized population. Synchronized L4 worms were transferred to fresh NGM plates seeded with the tested bacterium or *E. coli* OP50 or *E. coli* OP50 plus AFB1 or degradation metabolites every 2 days. Worms were considered dead when they stopped pharyngeal pumping and did not respond to prodding with a platinum wire. The number of dead/live worms was recorded every day.

### Ames Mutagenicity Assay

To evaluate the mutagenicity of the degradation metabolites, the *Salmonella* (Ames) test was conducted with the S9 Enzyme Activation kit (Iphase Pharma Service, Beijing, China) according to the manufacturer’s instructions and the procedure described by Fang et al. (2020). Briefly, the degradation metabolites extracted from a 96-h culture co-incubated with the AFB1-degrading bacterium and AFB1 were incubated with *Salmonella typhimurium* TA98 or TA100 at 37°C for 48 h. The number of *S. typhimurium* colonies was recorded, and the data were given as the number of reversed colony-forming units (CFUs). Samples extracted from LB medium with AFB1 were used as positive controls, and extracts from LB medium were used as negative controls.

### Aflatoxin B1 Degradation by Extracellular Extracts, Intracellular Extracts, and Dead Cells

The dilution of bacterial cells (OD_600_ = 0.02, the same below unless specified) was cultured in LB medium with shaking for 48 h at 37°C, and the supernatant and cells were collected, respectively, after centrifugation at 12,000 rpm for 5 min at 4°C. The supernatant filtered with a 0.22-μm pore filter served as the extracellular extracts for AFB1 degradation. After washing with 10 mM phosphate buffer (pH 8.0) three times, the cells were broken by ultrasonication (25 kHz, ultrasound for 4 s interval 1 s, 15 min) in the ice bath, and centrifuged at 12,000 rpm for 15 min at 4°C. The supernatant filtered with 0.22-μm pore filter served as the intracellular extracts for AFB1 degradation. Meanwhile, cells washed with phosphate buffer were boiled for 20 min, resuspended in an equal volume of 10 mM phosphate buffer (pH 8.0), and served as dead cells for AFB1 degradation. Extracellular extracts, intracellular extracts, and dead cells were co-incubated with 2 μg/ml of AFB1 at 37°C with shaking at 180 rpm for 72 h, respectively. Cultures of LB medium or phosphate buffer supplemented with 2 μg/ml AFB1 were used as the control, and all variables of control groups were similar to those of the corresponding extracts and dead cells. Residual AFB1 was tested as described above.

### Effect of Proteinase K, Sodium Dodecyl Sulfate, and Heat Treatment on Aflatoxin B1 Degradation

Extracellular extracts were divided into four fractions to investigate the influence of proteinase K, SDS, and heat on AFB1 degradation. One fraction was boiled for 20 min, and other fractions were treated with proteinase K (1 mg/ml), SDS (1%), or SDS plus proteinase K for 6 h, respectively. Subsequently, each fraction was incubated with 2 μg/ml AFB1 at 37°C with shaking at 180 rpm, and phosphate buffer with 2 μg/ml AFB1 was used as the control. After 24 h, residual AFB1 was monitored as described above.

### Effects of Aflatoxin B1 Concentrations, Temperature, pH Values, and Metal Ions on Aflatoxin B1 Degradation by the Aflatoxin B1-Degrading Bacterium and Its Cell-Free Supernatant

To investigate the effects of AFB1 concentrations on AFB1 degradation mediated by the AFB1-degrading bacterium, bacterial cells were incubated with 1, 2, 5, and 8 μg/ml AFB1, respectively, at 37°C for 96 h by shaking at 180 rpm. LB medium with the corresponding concentration of AFB1 was used as the control. The effects of temperature, pH, and metal ions were determined by setting the cultivation temperature at 25, 30, 37, 40, 45, or 50°C, adjusting the initial pH values to 5.0, 6.0, 7.0, 8.0, or 9.0, and adding MgSO_4_ (0.5 mg/ml), ZnSO_4_⋅7H_2_O (0.5 mg/ml), CuSO_4_⋅5H_2_O (0.5 mg/ml), MnSO_4_⋅H_2_O (0.5 mg/ml), FeSO_4_⋅7H_2_O (0.5 mg/ml), or CaCl_2_ (0.5 mg/ml). Bacterial cells were incubated in LB medium with 2μg/ml AFB1 at 37°C for 24, 48, or 72 h by shaking at 180 rpm. Correspondingly, LB medium with 2 μg/ml AFB1 in each incubation was used as the control. Residual AFB1 was detected by the HPLC described as above. In addition, bacterial growth was also investigated by measuring the OD_600_ value.

To investigate the effects of initial pH values, temperature, and metal ions on AFB1 degradation by the cell-free supernatant of AFB1-degrading bacterial culture, the cell-free supernatant was collected as described and exposed to 2 μg/ml of AFB1, and the mixture was incubated at 37°C by shaking at 180 rpm. The effects of initial pH values were analyzed by adjusting the mixture to 5.0, 6.0, 7.0, 8.0, or 9.0. In the temperature test, the mixture was incubated at 20, 30, 37, 40, 50, 60, or 70°C, respectively. In terms of metal ions, the reaction mixture was supplemented with 0.5 mg/ml of MgSO_4_, ZnSO_4_⋅7H_2_O, CuSO_4_⋅5H_2_O, MnSO_4_⋅H_2_O, FeSO_4_⋅7H_2_O, and CaCl_2_, respectively. Correspondingly, LB medium with 2 μg/ml of AFB1 in each incubation was used as the control. After a 48-h incubation, residual AFB1 was analyzed by HPLC as described above.

### Assays for the Fungal Growth and the Production of Aflatoxin B1 in *Aspergillus flavus*

To investigate an effect of the AFB1-degrading bacterium on the fungal growth of *A. flavus*, the antagonistic effect and dry weight were tested after the AFB1-degrading bacterium was co-incubated with *A. flavus* for 2 days in potato dextrose agar (PDA) and potato dextrose broth (PDB), respectively. About 1 μl of the bacterial cell dilution was spotted in the upper part of a PDA plate and 1 μl of a conidial suspension (1.0 × 10^6^ conidia/ml) of *A. flavus* was spotted in the lower part of the plate. The plates were incubated at 30°C for 2 days, and fungal growth was observed. Meanwhile, 100 μl of a conidial suspension (1.0 × 10^9^ conidia/ml) of *A. flavus* was added into 100 ml of PDB supplemented with bacterial cells (OD_600_ = 0.02) and incubated at 30°C by shaking at 180 rpm. PDB with *A. flavus* conidia was set as the control. After 2 days, fungal mycelia and the supernatant were collected by centrifugation. The collected mycelia were dried at 60°C and weighted. The supernatant was filtered by the 0.22 μm pore filter and analyzed by HPLC to detect the content of AFB1.

### Transcriptional Profiling of Genes Related to Aflatoxin B1 Synthesis

About 100 μl of a conidial suspension (1.0 × 10^9^ conidia/ml) of *A. flavus* was added to 100 ml of PDB supplemented with bacterial cells (OD_600_ = 0.02) and incubated at 30°C by shaking at 180 rpm. PDB with *A. flavus* conidia was set as the control. After 2 days, fungal mycelia were harvested and used to extract total RNA using the RNAiso™ Plus reagent (TaKaRa, Dalian, China). Total RNA was reversely transcribed to cDNA using the PrimeScript^®^ RT reagent kit (TaKaRa). Transcripts of targeted genes were quantified *via* real-time quantitative polymerase chain reaction (qRT-PCR) with paired primers (Supplementary Table 1) under the action of SYBR^®^ Premix Ex TaqTM (TaKaRa). The transcript of the fungal β*-*tublin gene was used as an internal standard. The relative transcript level of each gene was calculated as the ratio of its transcript in the group of *A. flavus* plus bacterial cells to the control group, using the threshold-cycle (2–ΔΔ*^Ct^*) method.

### Statistical Analysis

All the above experiments were conducted three times. The results of three replicates were expressed as mean ± standard deviation (SD), and statistical analysis was subjected to one-factor analysis of variance (ANOVA) performed with SPSS software. It is considered statistically significant when *p* < 0.05 in all the experiments.

## Results

### Isolation and Identification of Aflatoxin B1-Degrading Bacteria

**FIGURE 1:**
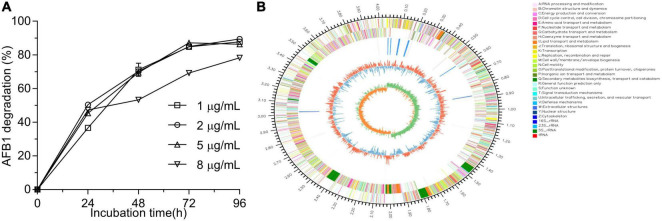
Aflatoxin B1 (AFB1) degradation mediated by *Bacillus amyloliquefaciens* WF2020 at different concentrations of AFB1 **(A)** and circular representation of the complete genome of *B. amyloliquefaciens* WF2020 **(B)**. From outermost to innermost circle: circle 1, genome size; circle 2, genes on forward strand; circle 3, genes on reverse strand; circle 4, rRNA and tRNA; circle 5, GC content; and circle 6, GC skew.

After primary screening using coumarin as the sole carbon source and secondary screening by addition of 2 μg/ml AFB1 in LB medium, four isolates showed the ability to degrade AFB1 after a 48-h incubation (Supplementary Figure 1A). Among the four isolates, WF2020, which was isolated from naturally fermented pickles, displayed the maximum degradation ability up to 70.22% (Supplementary Figure 1A). When AFB1 concentration was not more than 5 μg/ml, except those in the first 24 h, the percentages of degrading AFB1 for WF2020 during the 96-h incubation were nearly similar among the treatments at the same cultivation time and nearly reached the maximum at 72 h where the percentage of AFB1 degradation was more than 84% (Figure 1A). Moreover, when AFB1 concentration was up to 8 μg/ml, WF2020 could degrade AFB1 in a time-dependent manner, and a reduction of more than 75% was observed at 96 h (Figure 1A).

WF2020 is a Gram-positive bacterium with the typical colony characteristics of *Bacillus* sp. (Supplementary Figure 1B). According to genome sequences obtained using Illumina Hiseq and a PacBio system, the complete genome sequence of WF2020 comprises a 4,043,726 bp circular chromosome, consisting of 4,133 predicted genes, 27 rRNA genes, and 86 tRNA genes (Figure 1B). In addition, no plasmid was observed in the genome. Based on the sequence analysis of 16s rRNA and other 31 housekeeping genes, including *dnaG*, *frr*, *infC*, *nusA*, *pgk*, *pyrG*, *rplA*, *rplB*, *rplC*, *rplD*, *rplE*, *rplF*, *rplK*, *rplL*, *rplM*, *rplN*, *rplP*, *rplS*, *rplT*, *rpmA*, *rpoB*, *rpsB*, *rpsC*, *rpsE*, *rpsI*, *rpsJ*, *rpsK*, *rpsM*, *rpsS*, *smpB*, and *tsf*, the closest relative of WF2020 was *B. amyloliquefaciens* strain (Supplementary Figures 1C,D). Therefore, this isolate was termed *B. amyloliquefaciens* WF2020.

### The Active Component to Degrade Aflatoxin B1 in *Bacillus amyloliquefaciens* WF2020 and Its Characteristics

**FIGURE 2:**
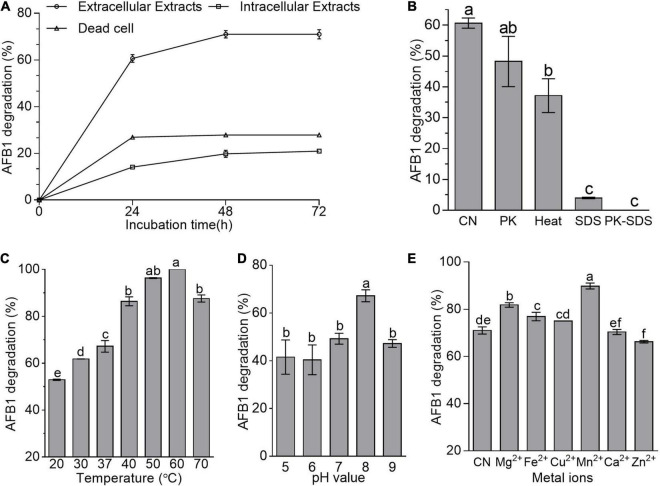
AFB1 degradation among diverse cell components of *B. amyloliquefaciens* WF2020. **(A)** AFB1 degradation by extracellular extracts, intracellular extracts, and dead cells during 72-h incubation with 2 μg/ml AFB1 at 37°C. **(B)** Effects of heat, proteinase K (PK), SDS, and proteinase K plus SDS on AFB1 degradation mediated by the cell-free supernatant after co-incubation for 24 h. **(C–E)** Effects of different temperatures **(C)**, pH values **(D)**, and metal ions **(E)** on AFB1 degradation mediated by the cell-free supernatant after co-incubation for 48 h. Different lowercase letters in the bars of each group indicate significant differences between treatments (Tukey’s test, *p* < 0.05).

Adsorption and degradation are the two main approaches in the removal of mycotoxins by microbes (Hathout and Aly, 2014). Here, cell-free supernatant (i.e., extracellular extracts) of *B. amyloliquefaciens* WF2020 was more effective than dead cells and intracellular extracts in reducing AFB1 during a 72-h incubation (Figure 2A). The percentage reduction of AFB1 for cell-free supernatant, intracellular extracts, and dead cells is 60.67, 14.11, and 26.95% at 24 h, 71.01, 19.80, and 27.87% at 48 h, and 71.01, 20.95, and 27.85% at 72 h (Figure 2A), respectively. Additionally, bacterial cells harvested from the cultivation of 48-h incubation in LB medium removed 12.25% of AFB1 on average after incubated with 2 μg/ml AFB1 for 1 h at 37°C by shaking at 180 rpm. These findings suggested that the removal of AFB1 mediated by *B. amyloliquefaciens* WF2020 was mainly dependent on the degradation and the cell-free supernatant was the main active ingredient during AFB1 degradation. Moreover, AFB1 degradation capacity of the cell-free supernatant decreased by 20.50, 93.40, and 100% after pretreatment with proteinase K, SDS, and SDS plus proteinase K (Figure 2B), respectively. Furthermore, cell-free supernatant still could degrade AFB1 by 37.16% after boiling for 20 min (Figure 2B). These results indicated that thermostable extracellular proteins or enzymes secreted by *B. amyloliquefaciens* WF2020 were involved in AFB1 degradation.

pH, temperature, and metal ions affected the AFB1 degradation ability of the cell-free supernatant from bacteria and fungi (Zhang et al., 2014; Rao et al., 2016; Wang et al., 2017; Xu et al., 2017; Shu et al., 2018). Here, AFB1 degradation studies with different incubation temperatures after 48-h incubation showed that the cell-free supernatant of *B. amyloliquefaciens* WF2020 could degrade AFB1 at temperatures ranging from 20°C to 70°C and the percentage of AFB1 degradation at 70°C remained more than 70% (Figure 2C), implying that the active constituents or components of the cell-free supernatant were thermostable and could work well within a wide range of working temperature. Moreover, the percentage of AFB1 degradation increased with the increase of temperature up to 60°C which was the optimum temperature for 100% AFB1 degradation (Figure 2C). In addition, the cell-free supernatant of *B. amyloliquefaciens* WF2020 could degrade AFB1 over a broad pH from 5.0 to 9.0 and the maximum percentage displayed at pH 8 (Figure 2D). Lastly, the effects of metal ions on the AFB1 degradation ability of the cell-free supernatant were evaluated (Figure 2E). Mn^2+^, Mg^2+^, Fe^2+^, and Cu^2+^ stimulated AFB1 degradation by 26.52, 15.19, 8.29, and 5.69%, respectively, whereas Ca^2+^ had no significant effect, but Zn^2+^ inhibited the degradation by 6.73% (Figure 2E), inferring that Mn^2+^, Mg^2+^, Fe^2+^, and Cu^2+^ may act as enzyme activators, membrane stabilizers, and help to maintain the structural integrity of proteins.

### Safety and Toxicity of *Bacillus amyloliquefaciens* WF2020 and Its Aflatoxin B1 Degradation Products

**FIGURE 3:**
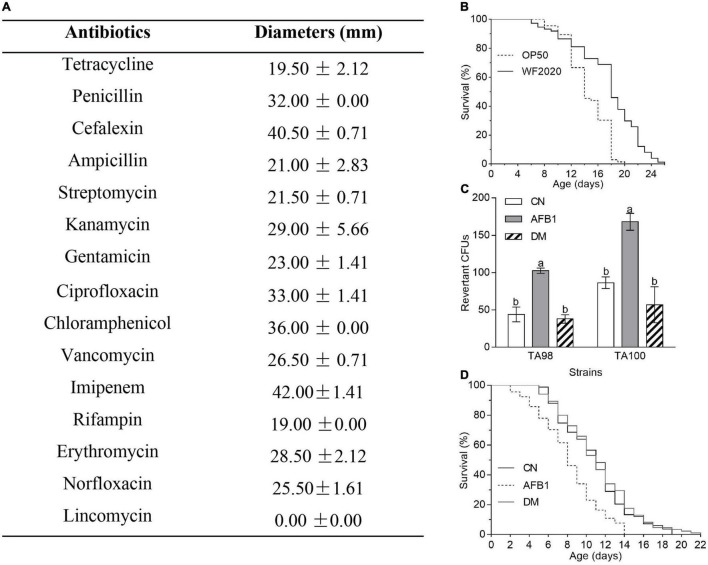
The safety of *B. amyloliquefaciens* WF2020 and its AFB1 degradation products. **(A)** Susceptibility of *B. amyloliquefaciens* WF2020 to different antibiotics by the disk diffusion test. **(B)** Changes in the lifespan of *C. elegans* N2 caused by *B. amyloliquefaciens* WF2020. **(C,D)** Reduction of AFB1 mutagenic effects **(C)** and the toxicity to *C. elegans* N2 **(D)** caused by *B. amyloliquefaciens* WF2020. The AFB1 group means extracts from the media supplemented with 20 μg AFB1. The DM group refers to the culture extracts from the supernatant of the 96 h co-incubation of 20 μg AFB1 and *B. amyloliquefaciens* WF2020. The CN group means the control group. Different lowercase letters in the bars of each group indicate significant differences between treatments (Tukey’s test, *p* < 0.05).

Based on the genomic sequence analysis, there are 12 secondary metabolic gene clusters *via* an antiSMASH analysis, but only six gene clusters harbored 100% similarity to those of known secondary metabolites (Table 1). The metabolites of the six gene clusters were macrolactin, bacillaene, fengycin, difficidin, bacillibactin, and bacilysin, respectively (Table 1), which are active substances with antibacterial, antifungal, anticancer, antiviral, anti-biofilm activities, biocontrol activity, etc. (Chen et al., 2008; Ryohei et al., 2015; Wu et al., 2015; Cochrane and Vederas, 2016; Zhou et al., 2018, 2021; Catherine et al., 2020; Erega et al., 2021; Kaushik et al., 2021). Moreover, a total of 35 genes with up to 50% similarity were found after blasting in the database of virulence factors, but they were not virulence genes but rather regulatory genes that played important roles in regulating biological processes, including virulence in other bacteria (Table 2). Additionally, a total of 19 genes with up to 50% similarity were found after blasting in the Comprehensive Antibiotic Resistance Database, and there is only one gene, *imrB* important for the resistance to lincosamide antibiotics, with up to 85% similarity (Table 3). Susceptibility to the corresponding antibiotics showed that, except for lincomycin belonging to a member of lincosamide antibiotics, *B. amyloliquefaciens* WF2020 was sensitive to other 14 antibiotics, including tetracycline, penicillin, cefalexin, ampicillin, streptomycin, kanamycin, gentamicin, ciprofloxacin, chloramphenicol, vancomycin, imipenem, rifampin, erythromycin, and norfloxacin (Figure 3A), suggesting their lower likelihood of being antibiotic-resistant bacterium.

**TABLE 1:** Secondary metabolites predicted by the antiSMASH analysis of *Bacillus amyloliquefaciens* WF2020.

Cluster type	MIBiG accession	Similarity	Location (Start-End)	Gene number
Surfactin	BGC0000433	82%	311953–377360	45
Butirosin	BGC0000693	7%	945967–987211	43
Macrolactin	BGC0000181	100%	1417655–1503557	46
Bacillaene	BGC0001089	100%	1734506–1837192	57
Fengycin	BGC0001095	100%	1909761–2047589	74
Difficidin	BGC0000176	100%	2356575–2457020	55
Bacillibactin	BGC0000309	100%	3099754–3166538	69
Bacilysin	BGC0001184	100%	3698104–3739520	45

**TABLE 2:** Genes with up to 50% similarity found in *B. amyloliquefaciens* WF2020 genome according to the database of virulence factors.

Gene ID	Annotation	Similarity
gene0118	ATPase	78.4%
gene0145	Elongation factor Tu	74.7%
gene0397	ABC transporter ATP-binding protein	51%
gene0654	Chaperonin GroEL	60.2%
gene0960	Catalase	55.9%
gene1113	Lipoate–protein ligase	61.6%
gene1492	ATP-dependent Clp protease ATP-binding subunit	62.6%
gene1684	Signal peptidase II	57.6%
gene1732	3-oxoacyl-[acyl-carrier-protein] reductase	50.4%
gene1733	Acyl carrier protein	63%
gene1766	Flagellar protein export ATPase FliI	52.9%
gene1775	Flagellar motor switch phosphatase FliY	51.7%
gene1778	Flagellar type III secretion system pore protein FliP	52.1%
gene1779	Component of the flagellar export machinery	52.9%
gene1796	Isoprenyl transferase	58.6%
gene2028	UTP–glucose-1-phosphate uridylyltransferase GalU	52.4%
gene2174	UDP-glucose 4-epimerase	51.2%
gene2196	Peptide-methionine (R)-*S*-oxide reductase MsrB	56.6%
gene2490	NADP-dependent phosphogluconate dehydrogenase	70.9%
gene2613	Superoxide dismutase	52.6%
gene3152	Conserved hypothetical protein	73.9%
gene3250	(2,3-dihydroxybenzoyl)adenylate synthase	55.1%
gene3361	ABC transporter ATP-binding protein	53%
gene3517	Polysaccharide biosynthesis protein	51.5%
gene3544	ATP-dependent Clp endopeptidase proteolytic subunit ClpP	77.9%
gene3661	UDP-*N*-acetylglucosamine 2-epimerase (non-hydrolyzing)	62.7%
gene3662	UTP–glucose-1-phosphate uridylyltransferase GalU	58.1%
gene3663	Teichoic acids export ABC transporter ATP-binding subunit TagH	55.7%
gene3687	Poly-gamma-glutamate biosynthesis protein PgsC	76.4%
gene3688	Poly-gamma-glutamate synthase PgsB	67.4%
gene3774	Urease subunit alpha	61.7%
gene3775	Urease subunit beta	50%
gene3853	Helix-turn-helix transcriptional regulator	50%
gene4023	UDP-glucose 4-epimerase GalE	64.7%

**TABLE 3:** Genes with up to 50% similarity found in *B. amyloliquefaciens* WF2020 genome according to the Comprehensive Antibiotic Resistance Database.

gene ID	*ARO name	Drug class	Similarity
gene0140	rpoB2	Peptide antibiotic, Rifamycin antibiotic	64.3%
gene0204	ampC1	Cephalosporin/Penam antibiotic	53%
gene0270	mphK	Macrolide antibiotic	64.8%
gene0281	lmrB	Lincosamide antibiotic	89.2%
gene0326	tmrB	Nucleoside antibiotic	77.2%
gene0596	vmlR	Lincomycin/Macrolide/Oxazolidinone/Phenicol/Pleuromutilin antibiotic	71%
gene0745	aadK	Aminoglycoside antibiotic	63.8%
gene0895	mprF	Peptide antibiotic	78.8%
gene1145	blt	Acridine dye, Fluoroquinolone antibiotic	77.6%
gene1191	fosB	Fosfomycin	63.5%
gene1263	bcII	Cephalosporin, Penam	52.6%
gene1306	bla1	Penam	63.7%
gene1414	tetA	Penam/Tetracycline antibiotic	52.5%
gene1425	ykkC	Aminoglycoside/Phenicol/Tetracycline antibiotic	79.5%
gene1426	ykkD	Aminoglycoside/Phenicol/Tetracycline antibiotic	81.7%
gene1913	rphB	Rifamycin antibiotic	78.7%
gene2207	dfrG	Diaminopyrimidine antibiotic	51.9%
gene2707	tet L	Tetracycline antibiotic	80.5%
gene2709	sat-4	Nucleoside antibiotic	52.2%

*ARO means Antibiotic Resistance Ontology.

Moreover, *C. elegans* has emerged as an invertebrate model to study host–pathogen interactions since its first documentation by Sydney Brenner (Brenner, 1974; Kumar et al., 2020). In the current study, *C. elegans* was used to evaluate the toxicity of *B. amyloliquefaciens* WF2020 to animals. *C. elegans* fed on *B. amyloliquefaciens* WF2020 cells showed significantly increased longevity compared with the effect of the laboratory-feeding bacterium *E. coli* OP50 cells when used as a food source (Figure 3B). The survival of worms fed on WF2020 cells increased by an average of 20.78% (average survival: 14.58 days, 95% confidence interval (CI): 13.83–15.32) compared with the strain OP50 (average survival: 17.61 days, 95% CI: 16.47–18.75). Maximum lifespans of worms fed on WF2020 were prolonged by 6 days compared with the strain OP50.

Except for the safety of the AFB1-degrading bacterium, the toxicity of AFB1 degradation products should not be neglected as some degradable products might be toxic like AFB1. In this case, *B. amyloliquefaciens* WF2020 might degrade AFB1 into C_15_H_11_O (m/z 207.08), C_15_H_15_O_2_ (m/z 227.11), and C_15_H_19_O_4_ (m/z 263.13), according to the HPLC-Q-TOF-MS analysis of the 72-h co-incubation culture of AFB1 and *B. amyloliquefaciens* WF2020 (Supplementary Figure 2), compared with those of AFB1 solution and the fermentation culture of *B. amyloliquefaciens* WF2020. Firstly, the Ames test was used to assess the mutagenicity of AFB1 degradation products by *B. amyloliquefaciens* WF2020. Compared with the control group, an approximately twofold increase in the number of revertant CFUs from *S. typhimurium* TA98 and TA100 was observed in the AFB1 group, but there was no significant difference in revertant CFUs of the degradation products and the control group (Figure 3C), indicating that *B. amyloliquefaciens* WF2020 converted AFB1 to the metabolites with a loss of mutagenicity. Except for mutagenicity, AFB1 decreased the lifespan and increased the mortality rate of *C. elegans* (Yang et al., 2015). Therefore, the effect of AFB1 and its degradation products on the lifespan of *C. elegans* was performed to further evaluate the toxicity of AFB1 degradation products mediated by *B. amyloliquefaciens* WF2020. The mean lifespan exposed to AFB1 significantly decreased by 25.14% compared with the control, but there was no significant difference in the survival rates of *C. elegans* exposed to degradation products and the control (Figure 3D), implying that AFB1 degradation products mediated by *B. amyloliquefaciens* WF2020 were not toxic to the lifespan of *C. elegans*. These findings demonstrated that *B. amyloliquefaciens* WF2020 degraded AFB1 into metabolites, which exhibited no mutagenicity or toxicity to the lifespan of *C. elegans*.

These collective results demonstrated that *B. amyloliquefaciens* WF2020 might be used as a potential probiotic to degrade AFB1 in food and feed.

### Effect of Fermentation Conditions on Aflatoxin B1 Degradation by *Bacillus amyloliquefaciens* WF2020

**FIGURE 4:**
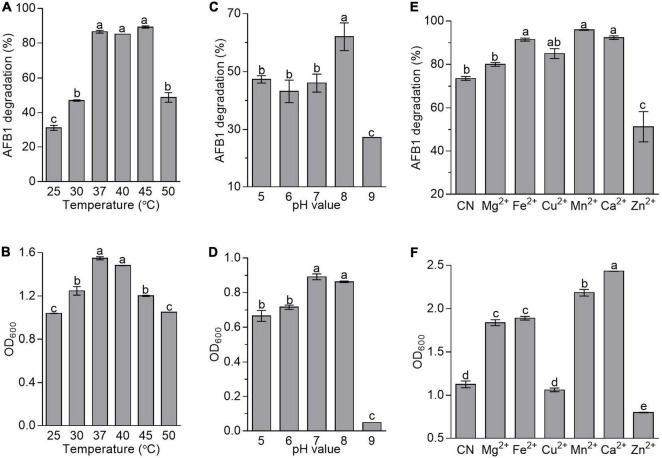
Effects of temperature **(A,B)**, initial pH value **(C,D)**, and metal ions **(E,F)** on the AFB1 degradation **(A,C,E)** and bacterial growth **(B,D,F)** in *B. amyloliquefaciens* WF2020. In terms of temperature, the residual AFB1 was analyzed after *B. amyloliquefaciens* WF2020 was co-incubated with 2 μg/ml AFB1 for 72 h. With respect to the effects of initial pH value and metal ions, the residual AFB1 was analyzed after *B. amyloliquefaciens* WF2020 was co-incubated with 2 μg/ml AFB1 at 37°C for 24 and 48 h, respectively. Different lowercase letters on the bars of each group indicate significant differences between the treatments (Tukey’s test, *p* < 0.05).

To evaluate the effects of fermentation conditions on AFB1 degradation mediated by *B. amyloliquefaciens* WF2020, incubation temperature, the initial pH of the culture, and metal ions were chosen as the tested fermentation conditions. In this study, AFB1 was degraded by *B. amyloliquefaciens* WF2020 at all incubation temperatures after 72-h incubation. The percentage of AFB1 degradation was 31.20, 46.99, 86.53, 85.16, 89.24, and 48.79% on average at 25, 30, 37, 40, 45, and 50°C, respectively (Figure 4A). However, the degradation rate showed no significant difference in the range of 37–45°C (Figure 4A). The growth of *B. amyloliquefaciens* WF2020 at 25, 30, 45, and 50°C decreased by 32.94, 19.47, 22.44, and 32.23%, respectively, compared with that at 37°C, and bacterial growth at 37 and 40°C showed no significant difference (Figure 4B). Combined with the effects of different temperatures on the AFB1 degradation ability of the active component of *B. amyloliquefaciens* WF2020, we speculated the lower degradation of AFB1 mediated by *B. amyloliquefaciens* WF2020 at 25 and 30°C might be due to the lower bacterial growth and lower activities of the active components at 25 and 30°C, and the lower degradation of AFB1 at 50°C might be attributed to the lower bacterial growth of *B. amyloliquefaciens* WF2020.

In *B. velezensis* DY3108, AFB1 degradation capability decreased in parallel with a decrease in initial pH (Shu et al., 2018), suggesting that the initial pH of the medium might be a critical factor in AFB1 degradation mediated by *Bacillus*. Here, AFB1 degradation mediated by *B. amyloliquefaciens* WF2020 was also sensitive to the initial pH of the medium. The percentage of AFB1 degradation was 47.36, 43.20, 46.08, 62.02, and 27.01% on average at an initial pH of 5, 6, 7, 8, and 9, respectively (Figure 4C), indicating that an initial pH value at 8.0 favored degradation when AFB1 was co-incubated with *B. amyloliquefaciens* WF2020. Moreover, the growth of *B. amyloliquefaciens* WF2020 at an initial pH of 5, 6, and 9 was inhibited by 25.36, 19.70, and 94.50%, respectively, compared with that at an initial pH of 7, and bacterial growth at an initial pH of 7 and 8 showed no significant difference (Figure 4D). Thus, the significant decrease in the percentage of AFB1 degradation mediated by *B. amyloliquefaciens* WF2020 at pH 9.0 might be due to the severe bacterial growth defects at pH 9.0.

With respect to the effect of metal ions on AFB1 degradation mediated by *B. amyloliquefaciens* WF2020, it was observed that, compared with the control, Mn^2+^, Ca^2+^, Fe^2+^, and Cu^2+^ stimulated degradation by 30.24, 25.35, 24.14, and 15.36%, respectively, and Mg^2+^ showed no significant difference though the percentage of AFB1 degradation increased by 8.61%, whereas Zn^2+^ inhibited degradation by 30.39% (Figure 4E). Moreover, compared with the control group, the growth of *B. amyloliquefaciens* WF2020 treated with Mg^2+^, Fe^2+^, Mn^2+^, and Ca^2+^ increased by 63.22, 67.84, 94.00, and 116.13%, respectively, but Zn^2+^ inhibited bacterial growth by 29.05% and Cu^2+^ had no significant effect on bacterial growth (Figure 4F). Combined with the effects of different metal ions on the AFB1 degradation ability of the active component of *B. amyloliquefaciens* WF2020, we speculated that changes in AFB1 degradation by *B. amyloliquefaciens* WF2020 caused by Fe^2+^, Mn^2+^, and Zn^2+^ might be due to the effects of corresponding metal ions on bacterial growth and active component capacities, and the increase in AFB1 degradation by *B. amyloliquefaciens* WF2020 caused by Ca^2+^ and Cu^2+^ might be attributed to the increase in bacterial growth and active component capacities caused by the corresponding metal ions, respectively.

### Effects of *Bacillus amyloliquefaciens* WF2020 on the Fungal Growth and Production of Aflatoxin B1 in *Aspergillus flavus*

**FIGURE 5:**
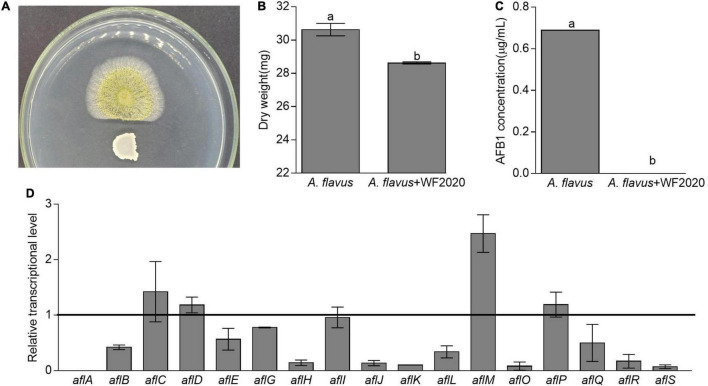
Changes in fungal growth, AFB1 production, and transcriptional expression of genes involved in AFB1 synthesis in *Aspergillus flavus*. **(A)** The antagonistic effect of *B. amyloliquefaciens* WF2020 on fungal growth after the bacterium was co-incubated with *A. flavus* for 2 days on a plate of potato dextrose agar (PDA). **(B,C)** The reduction of dry weight **(B)** and AFB1 production **(C)** caused by *B. amyloliquefaciens* WF2020 after the bacterium was co-incubated with *A. flavus* for 2 days in the potato dextrose broth (PDB). **(D)** Quantitation of relative transcriptional levels of selected genes associated with AFB1 synthesis in *A. flavus via* real-time quantitative polymerase chain reaction (qRT-PCR) after *B. amyloliquefaciens* WF2020 was co-incubated with *A. flavus* for 2 days in PDB. The line represents the transcriptional levels of genes in control experiments, which were defined as 1. Different lowercase letters in the bars of each group indicate significant differences between treatments (Tukey’s test, *p* < 0.05).

Except for AFB1 degradation, *B. amyloliquefaciens* WF2020 could inhibit the fungal growth of *A. flavus* and reduce AFB1 production. Pairwise interaction on agar plates proved that *B. amyloliquefaciens* WF2020 inhibited the fungal growth of *A. flavus* (Figure 5A). Moreover, the dry weight of the co-incubation culture of *B. amyloliquefaciens* WF2020 and *A. flavus* was reduced by 6.55% compared with that of *A. flavus* culture (Figure 5B). Additionally, *B. amyloliquefaciens* WF2020 completely inhibited AFB1 production when co-incubated with *A. flavus* in PDB for 2 days (Figure 5C). Accompanied by a reduction of AFB1 production, *B. amyloliquefaciens* WF2020 suppressed the transcriptional expression of 10 aflatoxin pathway genes (*aflA*, *aflB*, *aflE*, *aflG*, *aflH*, *aflJ*, *aflK*, *aflL*, *aflO*, and *aflQ*) and 2 gene encoding transcription factor *aflR* and *aflS* by 22.44–100% but increased the expression of *aflM*, an aflatoxin pathway gene, by 146.98% (Figure 5D). The downregulated expression of 10 aflatoxin pathway genes and 2 transcription factors suggested that AFB1 synthesis might be inhibited by *B. amyloliquefaciens* WF2020, which might result in reduced AFB1 production caused by *B. amyloliquefaciens* WF2020.

## Discussion

Generally, *B. amyloliquefaciens* was considered as a safe and non-toxic producing microbe and could be used for food and pharmaceutical purposes (WoldemariamYohannes et al., 2020). It was also reported that some strains of *B. amyloliquefaciens*, such as *B. amyloliquefaciens* UTB2, UNRC52, UNRCLR, S8C, Y1-B1, SWUN-TP23, SG-16, and HSP-5, could inhibit AFB1 synthesis or accelerate AFB1 degradation (Bluma and Etcheverry, 2006; Xu et al., 2015; Guo et al., 2017; Siahmoshteh et al., 2018; Wang J. et al., 2018; Ali et al., 2021; Zhang et al., 2021). Here, our results indicated that *B. amyloliquefaciens* WF2020 derived from naturally fermented pickles could act as a potential probiotic to efficiently detoxify AFB1 in a time dependent manner in ranges of 1–8 μg/ml and inhibit the fungal growth of *A. flavus* and AFB1 production, as discussed below.

Firstly, *B. amyloliquefaciens* WF2020 can degrade AFB1 ranging from 1 to 5 μg/ml by more than 80% after a 72-h incubation, which was similar to the 85.50% reduction of AFB1 at the concentration of 0.5 μg/ml reported in *B. amyloliquefaciens* SG16 (Wang J. et al., 2018) and was significantly higher than the 42.13 and 58.77% reduction reported in *B. amyloliquefaciens* SWUN-TP23 and HSP-5, respectively (Xu et al., 2015; Guo et al., 2017) and the 40 and 73.2% reduction of AFB1 at the concentration of 0.5 μg/ml reported in *B. amyloliquefaciens* S8C and Y1-B1, respectively (Ali et al., 2021; Zhang et al., 2021). Compared with the degradation abilities of AFB1 in the reported *Bacillus* species, the degradation ability in *B. amyloliquefaciens* WF2020 is similar to that in *B. licheniformis* BL010 (Wang Y. et al., 2018), *B. velezensis* DY3108 (Shu et al., 2018), and *B. subtilis* UTBSP1 (Farzaneh et al., 2012), higher than that in *B. subtilis* JSW-1 (Xia et al., 2017) but slightly lower than that in *Bacillus* sp. TUBF1 (El-Deeb et al., 2013) and *B. licheniformis* CFR1 (Rao et al., 2016), suggesting that there were great differences in degradation efficiency from one strain to other. Moreover, AFB1 degradation by *B. amyloliquefaciens* WF2020 was affected by fermentation temperatures, initial pH values, and metal ions. The temperature and initial pH value at the maximum degradation of AFB1 were 45°C and pH 8.0, respectively. Mn^2+^, Ca^2+^, Fe^2+^, and Cu^2+^ stimulated AFB1 degradation, and Mg^2+^ had no effect but Zn^2+^ inhibited the degradation. Compared with the reported *Bacillus* strains, the temperature was higher than the estimates of 30°C observed in *B. velezensis* DY3108 and 37°C observed in *B. cereus* CaG6 (Abdel-Shafi et al., 2018; Shu et al., 2018), and the pH value was the same to that observed in *B. velezensis* DY3108 (Shu et al., 2018). The stimulation induced by Ca^2+^ and the inhibition induced by Zn^2+^ were in agreement with the results in *Myroides odoratimimus* 3J2MO, but the stimulation induced by Fe^2+^, and Cu^2+^ and unchanged degradation caused by Mg^2+^ were opposite to the findings in *M. odoratimimus* 3J2MO (Mwakinyli et al., 2019). Meanwhile, the stimulation induced by Mn^2+^ was also opposite to that in *M. odoratimimus* 3J2MO (Mwakinyli et al., 2019) but was in well agreement with that in *B. cereus* CaG6 (Abdel-Shafi et al., 2018).

Secondly, the removal of mycotoxins by microbes was mainly attributed to adsorption and degradation (Hathout and Aly, 2014). In *B. amyloliquefaciens* WF2020, the removal of AFB1 was mainly dependent on degradation, and extracellular proteins or enzymes were the main active ingredient, which was similar to previous studies on AFB1 degradation mediated by *Bacillus*, such as *B. amyloliquefaciens* SG16 (Wang J. et al., 2018), *B. licheniformis* CFR1 (Rao et al., 2016), *B. subtilis* UTBSP1 and JSW-1 (Farzaneh et al., 2012; Xia et al., 2017), *B. velezensis* DY3108 (Shu et al., 2018), and *B. shackletonii* L7 (Xu et al., 2017). Moreover, the AFB1 degradation ability of extracellular proteins or enzymes was affected by temperature, the pH value, and metal ions. Increased temperatures may have promoted the bioavailability of organic compounds and facilitated biodegradation (Müller et al., 1998). Here, the percentage of AFB1 degradation mediated by the cell-free supernatant increased with the increase of temperature up to 60°C where 100% of AFB1 was removed, and the percentage of AFB1 degradation at 70°C remained more than 70%. Compared with the reported *Bacillus*, the thermostability of the cell-free supernatant of *B. amyloliquefaciens* WF2020 was similar to that from *B. shackletonii* L7 (Xu et al., 2017) and higher than that from *B. amyloliquefaciens* SG16 (Wang J. et al., 2018), *B. licheniformis* CFR1 (Rao et al., 2016), and *B. subtilis* UTBSP1 (Farzaneh et al., 2012), but slightly lower than that of *B. velezensis* DY3108 (Shu et al., 2018). Additionally, the cell-free supernatant of *B. amyloliquefaciens* WF2020 could still degrade AFB1 by 37.16% after boiling for 20 min, which was lower than that of *B. amyloliquefaciens* Y1-B1 (Zhang et al., 2021). These results demonstrated that extracellular proteins or enzymes were thermostable and could work well within a wide range of working temperature, which was helpful for application in food and feed processing and industry for AFB1 degradation. With respect to pH values, the optimal pH value of the cell-free supernatant from *B. amyloliquefaciens* WF2020 was 8.0, which was the same to that of extracellular enzymes from *E. coli* CG1061 (Wang et al., 2019), *Stenotrophomonas maltophilia* 35-3 (Guan et al., 2008), *B. shackletonii* L7 (Xu et al., 2017), and *B. velezensis* DY3108 (Shu et al., 2018) and was slightly higher than 7.5 reported in *B. amyloliquefaciens* SG16 (Wang J. et al., 2018). In addition, the AFB1 degradation ability of the cell-free supernatant from *B. amyloliquefaciens* WF2020 was increased by Mn^2+^, Mg^2+^, Fe^2+^, and Cu^2+^ and inhibited by Zn^2+^ but was not affected by Ca^2+^, inferring that Mn^2+^, Mg^2+^, Fe^2+^, and Cu^2+^ may act as enzyme activators, membrane stabilizers, and help to maintain the structural integrity of proteins. The enhancement of AFB1 degradation ability induced by Cu^2+^ and the inhibition of AFB1 degradation caused by Zn^2+^ were in agreement with the findings of extracellular enzymes or culture supernatant in *B. shackletonii* L7 (Xu et al., 2017), *B. licheniformis* CFR1 (Rao et al., 2016), and *B. velezensis* DY3108 (Shu et al., 2018). Cu^2+^ may take part in the redox reaction in electron transport, transferring an oxygen atom to the AFB1 substrate, and the oxidized AFB1 would then be hydrolyzed into non-toxic products (Xu et al., 2017). It has been reported that the inhibition of AFB1 degradation by Zn^2+^ might be due to the change in enzyme conformation caused by Zn^2+^, which resulted in decreased affinity of AFB1 (D’souza and Brackett, 1998). The stimulation of AFB1 degradation caused by Mg^2+^ was similar to that of *B. licheniformis* CFR1 (Rao et al., 2016), but opposite to that of *B. amyloliquefaciens* SG16 (Wang J. et al., 2018) and *B. shackletonii* L7 (Xu et al., 2017). The increase in AFB1 degradation induced by Mn^2+^ was opposite to that in *B. amyloliquefaciens* SG16 (Wang J. et al., 2018), *B. shackletonii* L7 (Xu et al., 2017), and *B. velezensis* DY3108 (Shu et al., 2018) and different from no obvious changes in *B. licheniformis* CFR1 (Rao et al., 2016). The increase of AFB1 degradation induced by Fe^2+^ was opposite to that in *B. amyloliquefaciens* SG16 (Wang J. et al., 2018) and *B. licheniformis* CFR1 (Rao et al., 2016).

Thirdly, the application of *B. amyloliquefaciens* WF2020 in AFB1 degradation was safe. On one hand, *B. amyloliquefaciens* WF2020 could act as a safe and non-toxic producing microbe. Based on the genomic sequencing analysis, *B. amyloliquefaciens* WF2020 produces several active compounds such as macrolactin, bacillaene, fengycin, difficidin, bacillibactin, and bacilysin and does not contain virulence genes and any plasmid. Additionally, *B. amyloliquefaciens* WF2020 is not an antibiotic-resistant bacterium due to susceptibility to various antibiotics, including tetracycline, penicillin, cefalexin, ampicillin, streptomycin, kanamycin, gentamicin, ciprofloxacin, chloramphenicol, vancomycin, imipenem, rifampin, erythromycin, and norfloxacin. Moreover, *B. amyloliquefaciens* WF2020 significantly enhanced the lifespan of *C. elegans* by an average of 20.78%, which was slightly lower than that of *B. amyloliquefaciens* EnB-alf1 isolated from alfalfa (*Medicago sativa* L.) seeds (Zhang et al., 2019). On the other hand, *B. amyloliquefaciens* WF2020 converted AFB1 into metabolites with a loss of mutagenicity and non-toxicity to the lifespan of *C. elegans*. The loss of mutagenicity was also observed in *Aspergillus oryzae* MAO103 and MAO104, *Aspergillus niger* RAF106, *B. licheniformis* CFR1, and *Rhodococcus erythropolis* (Alberts et al., 2006; Rao et al., 2016; Lee et al., 2017; Fang et al., 2020). The detoxification of AFB1 was mainly focused on the damage of the AFB1 toxic group of coumarin, which is a carcinogenic group, and bifuran nucleus, which are basic toxic structures (Xie et al., 2019). The loss of mutagenicity and the mortality rate of *C. elegans* suggested that *B. amyloliquefaciens* WF2020 might detoxify AFB1 into non-toxic compounds with the damage of coumarin and/or bifuran nucleus. These findings demonstrated that *B. amyloliquefaciens* WF2020 could act as a probiotic used to degrade AFB1 in food and feed.

Lastly, *B. amyloliquefaciens* WF2020 could slightly inhibit the fungal growth of *A. flavus*, completely reduce AFB1 production, and significantly suppress the expression of some important genes involved in the synthesis of aflatoxins, such as *aflA, aflB*, *alfE*, *alfG*, *alfH*, *alfJ*, *alfK*, *alfL*, *alfO*, *alfQ*, *alfR*, and *alfS.* The inhibition of the fungal growth of *A. flavus* was lower than that in *B. amyloliquefaciens* UNRC52, UNRCLR, and HSP-5, *Bacillus safensis* RF69, *Bacillus. sp*. RP103, and *Bacillus sp*. RP242 (Bluma and Etcheverry, 2006; Xu et al., 2015; Einloft et al., 2021). The reduction in AFB1 production was similar to that in *B. amyloliquefaciens* UTB2, *B. amyloliquefaciens* UNRC52, and *B. amyloliquefaciens* UNRCLR, but greater than that in *B. safensis* RF69, *Bacillus. sp*. RP103, and *Bacillus sp*. RP242 (Bluma and Etcheverry, 2006; Siahmoshteh et al., 2018; Einloft et al., 2021). *aflA, aflB*, *alfE*, *alfG*, *alfH*, *alfJ*, *alfK*, *alfL*, *alfO*, and *alfQ* are important aflatoxin pathway genes, which encode two fatty acid synthases, a norsolorinic acid ketoreductase, a P450 monooxygenase, an alcohol dehydrogenase, an esterase, versicolorin B synthase, a cytochrome P450 monooxygenase, *O*-methyltransferase B, and a P450 monooxygenase, respectively (Yu, 2012). *alfR*, encoding the positive-acting transcription factor, is required for the transcriptional activation of most, if not all, structural genes in the aflatoxin gene cluster, such as *aflB*, *alfE*, *alfG*, *alfH*, *alfJ*, *alfK*, *alfL*, *alfO*, and *alfQ* (Price et al., 2006; Yu, 2012). *alfS*, bidirectionally transcribed from *aflR*, is necessary for aflatoxin formation by regulating several aflatoxin pathway genes, such as *alfA* and *aflB* (Yu, 2012). Therefore, it was speculated that *B. amyloliquefaciens* WF2020 might inhibit AFB1 synthesis by downregulating the expression of *aflR*, *aflS*, and several important aflatoxin pathway genes. The reduction in AFB1 production might be attributed to the inhibition of fungal growth and AFB1 synthesis and AFB1 degradation caused by *B. amyloliquefaciens* WF2020.

## Conclusion

*Bacillus amyloliquefaciens* WF2020 could act as a potential probiotic with susceptibility to various antibiotics, the synthesis of several active substances, and beneficial effects on the lifespan of *C. elegans* to degrade AFB1 into non-toxic products over a wide pH range from 5 to 9 and the temperature from 25 to 50°C. Bacterial growth and AFB1 degradation ability of *B. amyloliquefaciens* WF2020 were also affected by metal ions, including Mg^2+^, Fe^2+^, Cu^2+^, Mn^2+^, Ca^2+^, and Zn^2+^. This degradation was mainly attributed to extracellular proteins or enzymes possessing a wide reaction temperature ranging from 20 to 70°C and pH ranging from 5 to 9, which will be helpful for their application in the harsh conditions during food and feed processing. Moreover, *B. amyloliquefaciens* WF2020 also could inhibit fungal growth, reduce AFB1 production, and downregulate the expression of several aflatoxin pathway genes and two transcription factors (*aflR* and *aflS*) in *A. flavus*. Therefore, *B. amyloliquefaciens* WF2020 and/or its enzymes or proteins in the supernatant are new promising agents to protect food and feed from AFB1 contamination. However, the structure of degradation products and the purification of enzymes or proteins merit further investigation to elucidate the mechanisms of AFB1 degradation mediated by *B. amyloliquefaciens* WF2020, which will be helpful to exploit the probable agents used in food and feed processing to reduce AFB1 contamination.

## Data Availability Statement

The datasets presented in this study can be found in online repositories. The names of the repository/repositories and accession number(s) can be found below: https://www.ncbi.nlm.nih.gov/, CP092778.

## Author Contributions

GC and QF designed and performed the experiments, analyzed the data, and prepared this manuscript. ZheL performed the experiments and revised this manuscript. CX and ZhiL analyzed the data. QZ and LW designed the experiments. XF contributed to the revision of this manuscript. JW contributed to the revision of this manuscript and overall support of this study. All authors contributed to the article and approved the submitted version.

## Conflict of Interest

CX and ZhiL are employed by Guangdong Moyanghua Grains and Oils Co., Ltd. The remaining authors declare that the research was conducted in the absence of any commercial or financial relationships that could be construed as a potential conflict of interest.

## Publisher’s Note

All claims expressed in this article are solely those of the authors and do not necessarily represent those of their affiliated organizations, or those of the publisher, the editors and the reviewers. Any product that may be evaluated in this article, or claim that may be made by its manufacturer, is not guaranteed or endorsed by the publisher.

## Footnotes

1
http://canu.readthedocs.io/en/latest/

2
http://ccb.jhu.edu/software/glimmer/index.shtml

3
ftp://ftp.ncbi.nlm.nih.gov/blast/executables/blast+/2.3.0/

4
https://github.com/bbuchfink/diamond

5
http://www.hmmer.org/

6
www.majorbio.com
