# Supplementary material for: Detoxification of Aflatoxin B1 by a Potential Probiotic Bacillus amyloliquefaciens WF2020
Source: Front Microbiol. 2022 May 10;13:891091. doi: 10.3389/fmicb.2022.891091 (PMC9127598; doi:10.3389/fmicb.2022.891091)
Supplement: Supplementary file 1 [file Data_Sheet_1.docx]

**Detoxification of Aflatoxin B1 by *Bacillus amyloliquefaciens* WF2020, a potential probiotic**

Guojun Chen^1#^, Qian’an Fang^1#^, Zhenlin Liao^1#^, Chunwei Xu^2^, Zhibo Liang^2^, Tong Liu^1^, Qingping Zhong^1^, Li Wang^1^, Xiang Fang^1^*, Jie Wang^1^*

^1^Guangdong Provincial Key Laboratory of Food Quality and Safety, Guangdong Provincial Key Laboratory of Nutraceuticals and Functional Foods, College of Food Science, South China Agricultural University, Guangzhou 510642, China

^2^Guangdong Moyanghua Grains and oils co.LTD, Yangjiang, 529535, China

*Corresponding author. E-mail: [wangjielangjing@126.com](mailto:wangjielangjing@126.com,)


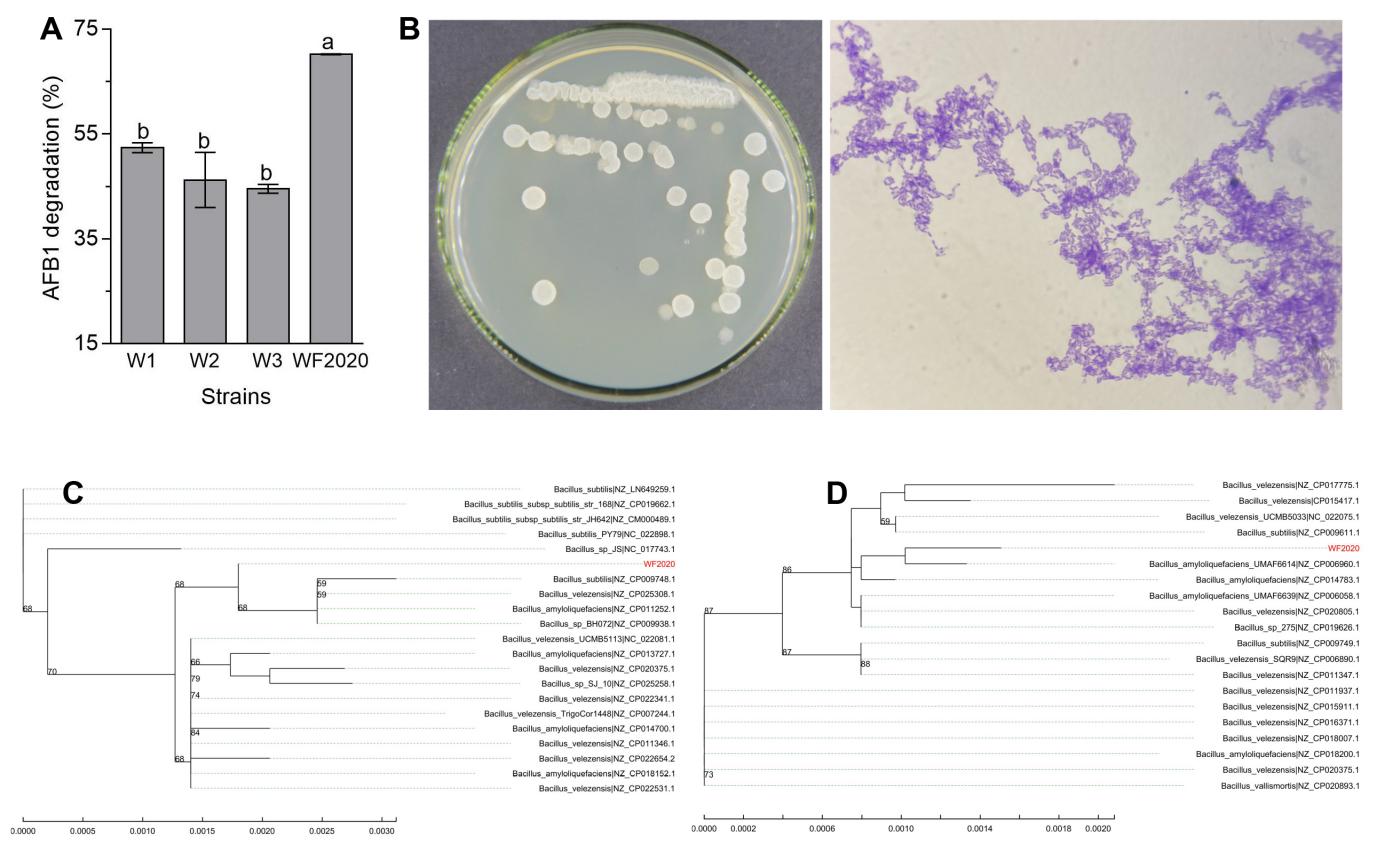


**Fig. S1** The AFB1 degradation and characteristics of bacteria from fermented foods. (A)The activities of AFB1 degradation mediated by four bacteria from fermented foods. (B) Images of the colonies and Gram staining of WF2020. (C-D)Phylogenetic trees for 16s rRNA (C) and 31 house-keeping genes (D) including *dnaG*, *frr*, *infC*, *nusA*, *pgk*, *pyrG*, *rplA*, *rplB*, *rplC*, *rplD*, *rplE*, *rplF*, *rplK*, *rplL*, *rplM*, *rplN*, *rplP*, *rplS*, *rplT*, *rpmA*, *rpoB*, *rpsB*, *rpsC*, *rpsE*, *rpsI*, *rpsJ*, *rpsK*, *rpsM*, *rpsS*, *smpB*, and *tsf* from the genome of WF2020 based on a neighbor-joining method, respectively. Scale bar: branch length proportional to genetic distance.


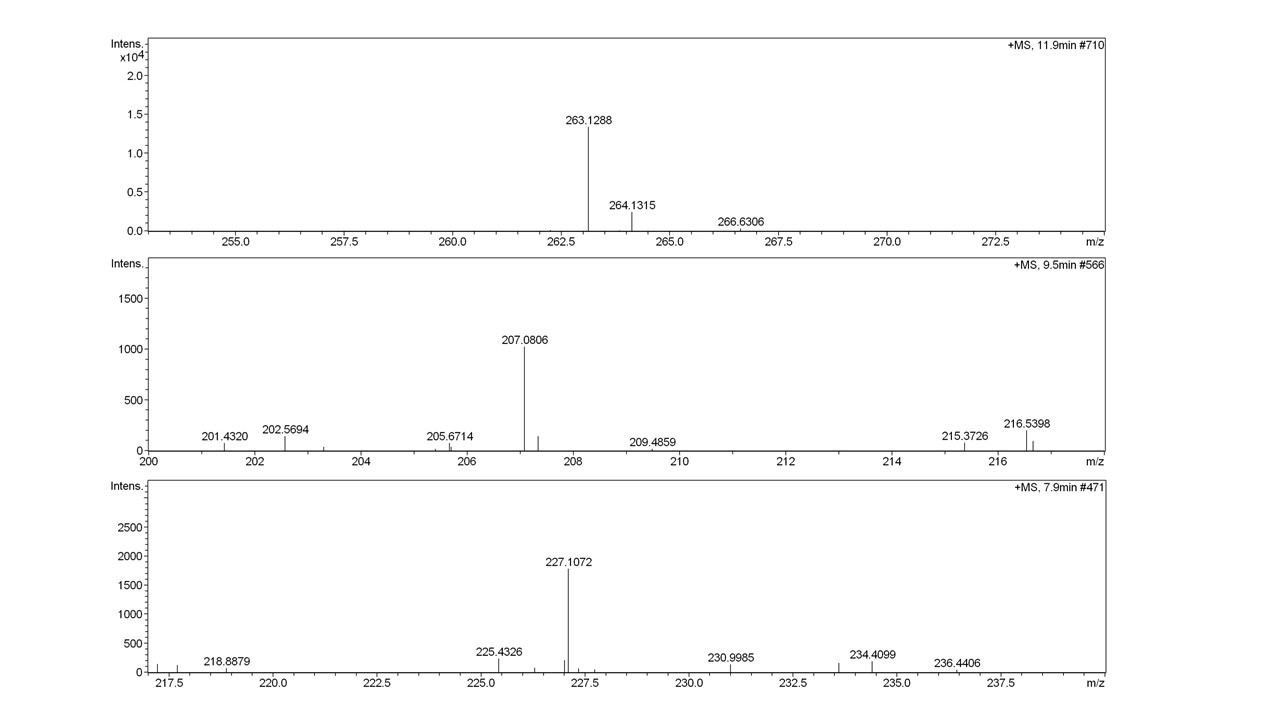


**Fig. S2** Mass spectra of potential degradation products for AFB1 by *B. amyloliquefaciens* WF2020

**Table S1.** Paired primers used for assessing transcript levels of 17 genes associated with the synthesis of AFB1 via qRT-PCR.

| **Gene** | **Annotation** | **Sequences (5'-3') of paired primers** |
| --- | --- | --- |
| *β-tublin* | Beta tublin | ATGGCTGCTTCTGACTTCCG/ CGCATCTGGTCCTCAACCTC |
| *aflA* | Fatty acid synthase alpha subunit | CCTATAAGTGCTTCAAAGATCGTGATCG/CGTACATGGATGACACGTTGTCCCAG |
| *aflB* | Fatty acid synthase subunit beta | TCTGATGGTCTCCGCTCTG/ TGTCCCTGACGCTGAATGT |
| *aflC* | Polyketide synthase | CCTATTCTAGCCGCCTTTCTTGAC/CATGTTGCCAGATTCCTCATATTCC |
| *aflD* | Norsolorinic acid reductase | TGTATGCTCCCGTCCTACTGTTTC/TGTAGTCTCCTTAGTCGCTTCATC |
| *aflE* | Norsolorinic acid ketoreductase | TCTAGCGCCGGTGTTCGT/ TTACCCCTTTCCAGCCATTG |
| *aflG* | P450 monooxygenase | GCGATAGAACTGACAAAGGCA/ GAATGAGTCTCCAAAGGCGAG |
| *aflH* | alcohol dehydrogenase | CCAGCTCCACGGTCGG/ CTTAAGGTCAAAGATTCCCTCGG |
| *aflI* | Oxidase | GATCGGCTCGTTTGAGGGA/GCAAAAATGATATTCAGCTGGTTTGAC |
| *aflJ* | Esterase | GCTCCGTGGCACCAGTT/ TCGTCACGCTCTCATCGG |
| *aflK* | VERB synthase | GAACTGCTTCAGTTGCCGTG/ ACGAGGGTTCGTTTCTGGAC |
| *aflL* | P450 monooxygenase | GATGCACCATGACCTCATGCGTTA/CACGGCAGCGTTATTGATCATCTC |
| *aflM* | Dehydrogenase | GCGGAGAAAGTGGTTGAACAGATC/CAGCGAACAAAGGTGTCAATAGCC |
| *aflO* | O-methyltransferase B | CTTTCGGCAGTGACCTAACC/ TCTTGAACTATAAGGCGACCAG |
| *aflP* | O-methyltransferase A | CGATGTCTATCTTCTCCGATCTATTC/TCTCAGTCTCCAGTCTATTATCTACC |
| *aflQ* | P450 monooxygenase | GGTTTTGACAGTGTCCGCAGTG/ AGAAGAGCACCTTTGGGCAG |
| *aflR* | Transcription factor | GCAACCTGATGACGACTGATATGG/TGCCAGCACCTTGAGAACGATAAG |
| *aflS* | Transcription factor | AAGCTAAGGCCGAGTCTGG/ CAGGTTGTGTTGCTGTTGATAG |
